# Safety and short term outcomes of a new truly minimally-invasive mesh-less and dissection-less anchoring system for pelvic organ prolapse apical repair

**DOI:** 10.1590/S1677-5538.IBJU.2016.0356

**Published:** 2017

**Authors:** Adi Y. Weintraub, Masha Ben Zvi, David Yohay, Joerg Neymeyer, Yonatan Reuven, Menahem Neuman, Alex Tsivian

**Affiliations:** 1 Department of Obstetrics and Gynecology, Soroka University Medical Center, Faculty of Health Sciences, Ben-Gurion University of the Negev, Beer Sheba, Israel;; 2 Department of Urologic Surgery, Edith Wolfson Medical Center, Holon, Sackler Faculty of Medicine, Tel Aviv University, Tel Aviv, Israel;; 3 Department of Urology, Charitè University, Berlin, Germany;; 4 Siaal Research Center for Family Medicine and Primary Care, Division of Community Health, Faculty of Health Science, Ben-Gurion University of the Negev, Beer Sheva, Israel;; 5 Urogynecology, Department of Obstetrics and Gynecology, Galilee Hospital, and the Faculty of Medicine in the Galilee, Bar Ilan University, Safed, Israel;; 6 Assuta Medical Centers, Tel Aviv and Rishon Le-Zion, Israel

**Keywords:** Prolapse, Minimally Invasive Surgical Procedures, Pelvic Floor Disorders

## Abstract

**Objective:**

To evaluate the safety and short term outcomes of a new, truly minimally-invasive, mesh-less and dissection-less anchoring system for pelvic floor apical repair.

**Methods:**

A prospective study was conducted using the NeuGuide™ device system for pelvic floor apical repair. The primary effectiveness outcome was centro-apical pelvic floor prolapse by POP-Q after six months. The primary safety outcome was intra-operative, immediate (first 48 h) post-operative complications and adverse effects after six months. A standardized questionnaire (UDI-6) to assess quality of life at entry and during follow-up visits was used. Patients’ six months-follow-up and evaluation are reported.

**Results:**

The mean age of the study population (n=10) was 63.8±12.0 years. All patients had a previous prolapse surgery. Five had a previous hysterectomy and two had stress urinary incontinence symptoms. During surgery six patients had a concurrent colporrhaphy. There was no injury to the bladder, rectum, pudendal nerves, or major pelvic vessels and no febrile morbidity was recorded. At six months, no cases of centro-apical recurrence were noted. Patients were satisfied with the procedure and had favorable quality of life scores. Using the UDI-6 questionnaire an improvement, in all domains was seen. Moreover, although the sample size was small, the improvement in urge and overflow incontinence related domains were demonstrated to be statistically significant.

**Conclusions:**

This new NeuGuide™ device allows rapid and safe introduction of a suspending suture through the sacrospinous ligament and makes sacrospinous ligament fixation easy to perform, while avoiding dissection and mesh complications.

## INTRODUCTION

Pelvic organ prolapse (POP) is a common problem in women and often requires surgical correction. In the USA, about 200,000 women undergo surgery for prolapse correction every year (1). The lifetime risk of a woman to undergo a surgical procedure for the correction of pelvic floor dysfunctions (PFD) is 11%. Among these women, there is a close to 30% risk of re-operation due to failure or prolapse of another compartment (2).

Apical prolapse is defined as the descent of the apex of the vagina into the lower vagina, to or beyond the hymeneal ring. The apex can be either the uterus and cervix, cervix alone, or vaginal vault, depending upon whether the woman has undergone hysterectomy. The classification of prolapse according to the separate compartments is arbitrary, since the vagina is a continuum and prolapse of one compartment is often associated with prolapse of another (3).

Loss of apical support is usually present in women with advanced and symptomatic prolapse that extends beyond the hymen. Women may present with symptoms of anterior, posterior, central prolapse or any combination of these. Clinical manifestations include a bulge sensation or vaginal pressure, urinary, defecatory or sexual dysfunction (4). There is growing understanding that adequate vaginal apex support is essential for a durable surgical repair in women with advanced prolapse (5). Moreover, surgical correction of the anterior and posterior walls may fail unless the apex is adequately supported (6).

There is a wide variety of surgical treatments available for prolapse; this indicates that there is a lack of consensus as to the optimal surgical approach (5, 7).

Transvaginal sacrospinous ligament fixation (SSLF) was shown to have shorter operating time, less wound complications, quicker recovery to daily activities, and was cheaper than abdominal sacrocolpopexy (8). Moreover, the vaginal approach facilitates the concomitant correction of other vaginal defects as well. Because of a high risk for ureteral injury, the sacrospinous ligament (SSL) is preferred over the uterosacral ligament as the fixation point (9). However, transvaginal anchoring or placement of the fixation sutures through a deep, narrow space to the SSL is technically challenging and potentially dangerous. Indeed, numerous surgical adjuncts for SSL anchoring or suture placement have been introduced over the years with no device proven to be superior to others (10-16). These techniques all require deep vaginal dissection in order to gain safe access to the SSL. Many SSLF operations involving mesh implants were criticized by the FDA as having increased risk for severe and frequent adverse effects (17).

We have recently published our preliminary study demonstrating the biomechanical properties, feasibility and potential advantages of a new device - the NeuGuide™ (18). This new anchoring device intends to provide a truly minimally invasive, dissection-less approach for SSLF. This device enables the surgeon to perform a pelvic centro-apical support operation with no mesh implants, using just suturing materials. The aim of the current prospective pilot study is to evaluate the safety and short term outcomes of this new anchoring system for pelvic floor apical repair.

## MATERIALS AND METHODS

### Study design

A prospective study was conducted, following IRB approval. Surgeries were carried out by two experienced and well-trained urologic surgeons. All surgeries were performed using the same surgical technique. The primary effectiveness outcome was centro-apical pelvic floor prolapse by measuring the POP-Q point C/D after 6 months. The primary safety outcome was intraoperative complications and adverse effects after 6 months. A standardized questionnaire to assess quality of life (QoL) at entry and during follow-up visits was used.

### Study population

A pilot of ten patients who presented with a diagnosis of POP-Q stage III centro-apical pelvic floor prolapse with significant symptoms were offered to enroll in the study. Informed consent was obtained after thorough information was presented. Inclusion criteria included: women aged 50-80 years, POP-Q stage III centro-apical pelvic floor prolapse, scheduled to undergo a POP surgery and had agreed to undergo it using the NeuGuide^TM^ device, and who were willing to return for follow-up evaluation and fill questionnaires as indicated by the study protocol. Women with a diagnosis of reproductive tract anomalies, prior pelvic radiation therapy or any malignancy, women with a significant history of pelvic inflammatory disease, women with a known allergy to Nickel or Nitinol and women unable to complete written questionnaires were excluded from the study.

### Data collection

Follow up assessment was carried out 4-6 weeks, three months and six months after surgery. We present the cumulative data regarding adverse outcomes at six months due to the small number of participants in this pilot study and the few events that occurred in order to avoid repetition and confusion. The UDI-6 score was provided for all follow up visits (n=8/10, n=6/10, n=9/10, respectively). Outcome measures included anatomical and functional cure rates, levels of post-operative pain and dyspareunia as well as intra and post-operative complication rates. Data was collected prospectively and included demographic features and validated PFD related quality of life (QoL) questionnaires (Urogenital Distress Inventory - UDI-6). Modified POP-Q scores (Ba, Bp, C and D) were measured preoperatively and at each post-operative visit. Stage of prolapse was defined as the most prolapsed compartment. Success of the operation was defined as a composite of no central compartment bulge symptoms and no prolapse beyond the stage I (1 cm proximal to the hymenal ring).

### Device description and surgical technique

The NeuGuide™ is designed to enable centro-apical pelvic floor support for the uterine cervix or vaginal vault without need of either vaginal dissection or mesh implants in patients with a central compartment defect that need suspension. The NeuGuide™ device is comprised of two main elements: an anchor unit and a delivery system. The delivery system enables the guidance, insertion and deployment of the anchor element. The device’s anchor unit is designed as a sharp needle point Nitinol harpoon enabling piercing through the vaginal layers and the ligament. The anchor is deployed and placed with the use of an applicator. The anchor incorporates a surgical suture at its distal end, which following its deployment enables fixation and the continuation of the surgical procedure as intended for the repair process. It has a thimble that is an accessory to the device and can be used as an introducer for better handling of the NeuGuide™.

The anchor penetration diameter is 2.0 mm. Once deployed (passed the SSL), the wings open to 4.0 mm. The work channel length is 120 m (this limits the anchor penetration depth beyond the ligament in order to avoid injury). The device shaft diameter is 2.5 mm and its length 285 mm. The suture length is 70 cm and the work channel is designed to fit all sizes (self-adjusting). The applicator includes two concentric hollow shafts. The outer shaft constrains the anchor wings from being deployed. Once the button is pressed, the inner shaft pushes the anchor distally and allows the wings to deploy. The applicator is equipped with a safety latch that protects the button, to avoid undesired deployment.

The steps of the surgical procedure are presented in Table-1.

**Table 1:** The steps of the NeuGuide device surgical procedure.

1	The NeuGuide device is mounted on the right index finger, and introduced into the vaginal cavity
2	The right iscial spine and the SSL are palpated through the vaginal wall
3	The index finger is stabilized intimately to the mid SSL
4	The anchor is deployed, and adequate pull-out force is proven
5	A 1 cm longitudinal shallow and high mucosal incision is made at the posterior vaginal wall
6	The anchor's suture is mounted on a virgin needle
7	The suture is inserted backwards through the vaginal wall at its entering point, passed under the vaginal wall, then through the cervical istmus and out to the vaginal cavity again through the posterior colpotomy
8	The previous steps are repeated on the left side and the suture is tied appropriately
9	The small posterior vaginal incision is closed

**SSL=** Sacro-spinous ligament

### Statistical analysis

All statistical analyses were performed using the Statistical Package for the Social Sciences (SPSS, software version 22.0). Data on continuous variables with normal distribution were presented as mean±SD. Ordinal variables were presented as median and range, statistical analysis was completed using the Wilcoxon test. Categorical data were shown in counts. Two-sided p-value of <0.05 was considered significant.

## RESULTS

Baseline preoperative clinical characteristics of the patients who underwent NeuGuide™ surgery are presented in Table-2. The mean age of the study population at the time of surgery was 63.8±12.0 years. All patients had a previous POP surgery, five had a previous hysterectomy and 2 had stress urinary incontinence (SUI) symptoms. All patients had at least stage II prolapse in at least two compartments. Preoperative point C/D POPQ showed a median (range) of 3 [2-4]. During surgery 6 patients had a concurrent colporrhaphy and no injury to the bladder, rectum, pudendal nerves, or major pelvic vessels were noted.

**Table 2:** Preoperative demographic and clinical characteristics of 10 patients who underwent NeuGuide™ surgery.

Characteristics	n = 10
Age	63.8±12.0
Body Mass Index	27.1±3.7
Parity	2 (1-3)
Health problems	6
SUI	2
Previous hysterectomy	5
Previous POP surgery	10
POP	10
Point C/D POPQ median (range)	3 (2-4)
Central compartment prolapse stage ≥ 3	10
Cystocele stage ≥ 2	9
Rectocele stage ≥ 2	7
Enterocele stage ≥ 2	0
Concomitant procedure	6
Anterior colporrhaphy	4
Posterior colporrhaphy	2
MUS	0

Values are presented as mean±SD, median and range or number of women

**MUS =** Midurethral sling; **SUI =** Stress urinary incontinence; **POP =** Pelvic organ prolapse; **POPQ =** Pelvic organ prolapse quantification system.

Table-3 presents postoperative outcomes of patients who underwent NeuGuide™ surgery. No post-operative febrile morbidity was recorded. At the six months-follow-up, none of the patients had prolapse symptoms. Patients were found to be satisfied with the procedure with a median of 9, and favorable QoL scores were recorded with a median of 8 (on a scale of 0=not at all to 10=very much) (Table-3). All patients had significantly improved anatomical results (median point C/D POPQ score 0).

**Table 3:** – Postoperative outcomes of patients who underwent NeuGuide™ surgery.

Characteristics	n = 10
Point C/D POPQ	-5 (-4/-7)
Hematoma formation	0
Abscess formation	0
Post-operative pelvic pain	0
Post-operative buttock pain	1
*De Novo* dyspareunia	0
*De Nov*o SUI	0
*De Novo* urinary frequency	0
*De Nov*o urge incontinence	1
Recurrent POP	0
Satisfaction	8 (9-7)
Quality of life	9 (9-7)

Values are presented as median and range or number of women.

**SUI =** Stress urinary incontinence; **POP =** Pelvic organ prolapse; **POPQ =** Pelvic organ prolapse quantification system.

Using the UDI-6 standardized questionnaire, all domains showed an improvement in symptom related QoL. Moreover, improvement in urge and overflow incontinence related domains was demonstrated to be statistically significant (Table-4).

**Table 4:** – Urogenital symptoms and Quality of life assessed with Urogenital Distress Inventory (UDI-6) (N=10).

UDI-6	Pre-surgery (N=10)	4-6 weeks after surgery (N=8)	Three months after surgery (N=6)	Six months after surgery (N=9)
Frequent urination	70	56*	50*	60
Urine leakage related to urgency	33	29	27	26*
Urine leakage related to physical activity	33	29	22	26
Small amounts of urine leakage (drops)	33	17	17	20*
Difficulty emptying your bladder	50	29	39	26
Pain or discomfort in the lower abdomen/genitalia	50	28	33	30

Values are presented as percentages.

*P value <0.05

## DISCUSSION

In this pilot study, we report our first short-term results of apical prolapse correction using a novel device - NeuGuide™ - a new anchoring device intended to provide a truly minimally invasive, dissection-less approach for SSLF. Our findings demonstrate that the primary effectiveness outcome, centro-apical pelvic floor prolapse at six months-follow-up, was highly successful. With regard to the primary safety outcomes, no intraoperative complications were recorded and after six months low complication rates were noted. None of the patients suffered from recurrent prolapse at the six months-follow-up. Satisfaction and QoL scores were high.

There is a wide variety of surgical procedures available for apical prolapse repair. This indicates that there is a lack of consensus as to the optimal surgical approach (5, 7). For years, mesh augmentation was the most common technique for apical prolapse repair. Following the FDA notification in 2011 (16), due to high occurrence of late complications with mesh as a foreign body, the current recommendation of the American College of Obstetricians and Gynecologists and the American Urogynecologic Society is for a judicious use of mesh implants that should be reserved for high risk individuals and selected patients only (19, 20).

POP is common and often requires surgical correction. The final surgical decision must be individualized. If a vaginal approach is chosen and the patient is sexually active, then anatomic preservation of the vagina should be pursued (21). This has traditionally been accomplished with SSLF or other vaginal procedures such as uterosacral ligament suspension (USLS). USLS may be easier to perform than SSLF, with less risk of hemorrhage or infection, but does carry a higher risk of ureteral injury especially in patients with concomitant anterior colporrhaphy (22). Moreover, USLS is less practical in treating patients with post-hysterectomy vault prolapse. In our study, five patients were post hysterectomy and six had a concomitant anterior or posterior prolapse repair.

Informed decision-making about optimal surgical repair of apical prolapse with vaginal native tissue versus transvaginal mesh requires understanding the balance between the potential “harm” of mesh-related complications and the potential “benefit” of reducing prolapse recurrence. Dieter et al. (23) examined this harm/benefit balance and concluded that based on the best available evidence, there is considerable uncertainty about the harm/benefit trade-off between native tissue and transvaginal mesh for apical prolapse repair (23). In our study using the NeuGuide™ device, mesh complications were taken out of the equation.

Recurrent prolapse is also a major concern following POP surgery. In our study, at six months, all patients had significantly improved anatomical results (median point C/D POPQ score 0) and no cases of recurrence were noted. Lavelle et al. (24) reported their experience with POP recurrence after native tissue anterior vaginal suspension procedures. After a mean follow-up at 5.8±3.7 years, they reported prolapse recurrence rates of approximately 45% (isolated anterior 7.4%, isolated apical 10.7%, isolated posterior 8.3%, multiple compartments 19%) (24). Compared to their results, ours seem promising. However, a long-term follow-up will be needed in order to establish the sustainability of these results.

The primary limitations of the study include its single-arm evaluation. The lack of a control group restricts the external validity of this study. The 6-months evaluation period also may not be long enough to draw substantial conclusions. Another limitation that could restrict the external validity of this study was the fact that all surgical procedures were performed by two surgeons (MN and AT) who have extensive experience with pelvic surgery. However, since this is a new procedure it is unlikely that the surgeon’s experience has affected the results. Finally, our study was too small to add meaningful data to the literature about objective and subjective outcomes of this device but it was a pilot study on human subjects and the outcomes are encouraging.

The strengths of the study include the prospective design allowing comprehensive data collection, the evaluation of self-reported patients centered outcomes and the use of validated QoL questionnaires. In addition, safety and efficacy of this new device were previously shown in a cadaver and animal study that was methodologically meticulous (18).

In conclusion, this new NeuGuide™ device allows rapid and safe introduction of a suspending suture through the SSL and makes SSLF easy to perform, while avoiding dissection and mesh complications. This procedure might be appropriate for patients with loss of apical support or elongated coli who wish to avoid mesh augmentation. Further studies are needed in order to substantiate these results and to increase the external validity of our findings.
